# Nutritional Management of Pediatric Gastrointestinal Motility Disorders

**DOI:** 10.3390/nu16172955

**Published:** 2024-09-02

**Authors:** Lucy Jackman, Lauren Arpe, Nikhil Thapar, Anna Rybak, Osvaldo Borrelli

**Affiliations:** 1 Neurogastroenterology & Motility Unit, Gastroenterology Department, Great Ormond Street Hospital for Children, London WC1N 3JH, UK; lucy.jackman@gosh.nhs.uk (L.J.); lauren.arpe@gosh.nhs.uk (L.A.); anna.rybak@gosh.nhs.uk (A.R.); 2 Department of Paediatric Gastroenterology, Hepatology and Liver Transplant, Queensland Children’s Hospital, School of Medicine, University of Queensland, Centre of Children Nutrition Research, Queensland University of Technology, Brisbane, QLD 4000, Australia; nikhil.thapar@health.qld.gov.au

**Keywords:** motility disorders, gastroesophageal reflux, atresia, achalasia, gastroparesis, pediatric intestinal pseudo-obstruction, nutritional management, enteral nutrition, parenteral nutrition

## Abstract

Normal and optimal functioning of the gastrointestinal tract is paramount to ensure optimal nutrition through digestion, absorption and motility function. Disruptions in these functions can lead to adverse physiological symptoms, reduced quality of life and increased nutritional risk. When disruption or dysfunction of neuromuscular function occurs, motility disorders can be classified depending on whether coordination or strength/velocity of peristalsis are predominantly impacted. However, due to their nonspecific presenting symptoms and overlap with sensory disruption, they are frequently misdiagnosed as disorders of the gut–brain interaction. Motility disorders are a prevalent issue in the pediatric population, with management varying from medical therapy to psychological therapy, dietary manipulation, surgical intervention or a multimodal approach. This narrative review aims to discuss the dietary management of common pediatric motility disorders including gastroesophageal reflux, esophageal atresia, achalasia, gastroparesis, constipation, and the less common but most severe motility disorder, pediatric intestinal pseudo-obstruction.

## 1. Introduction

The integration of digestive, absorptive, and motility functions within the gastrointestinal (GI) tract is pivotal for maintaining optimal nutrition. In a healthy gut, the enteric nervous system coordinates sensation and the rhythmic contractions necessary to propel food through the GI tract. However, disruption of this complex system can lead to undesirable symptoms, malabsorption and increased nutritional risk.

Among the most prevalent issues in the pediatric population are motility disorders of the GI tract and disorders of gut–brain interaction (DGBIs). Motility disorders encompass dysfunction characterized by impaired coordination, strength and/or velocity of peristalsis, and sensation often stemming from neuropathic or myopathic origins [1]. Conversely, DGBIs typically lack obvious structural or biochemical abnormalities, with reliance on clinical symptoms for their diagnosis. While the latter conditions may significantly impact quality of life (QOL), they are generally benign.

Despite differing pathologies, both conditions present with nonspecific symptoms such as constipation, reflux, abdominal pain, distension, nausea, and vomiting. Consequently, motility disorders are frequently misdiagnosed as DGBI, leading to delays in accurate diagnosis.

Management approaches encompass a spectrum of interventions, including medical therapies, surgical procedures, psychological interventions, dietary adjustments, or a multimodal approach, if required.

Herewith, we present a review of a dietary management of common pediatric motility disorders including gastroesophageal reflux, esophageal atresia, achalasia, gastroparesis and the less common but most severe motility disorder, pediatric intestinal pseudo-obstruction.

## 2. Materials and Methods

A literature search was conducted using EMBASE, PubMed, Google Scholar databases and Ovid to investigate nutritional management of various motility conditions. There was also a search conducted using specific keywords such as ‘paediatric/pediatric’, ‘children’, ‘gastroesophageal reflux’, ‘achalasia’, ‘atresia’, ‘gastroparesis’, ‘chronic intestinal/bowel pseudo-obstruction’, ‘enteral/parenteral nutrition’, ‘motility disorders’, ‘parenteral/enteral nutrition’, ‘jejunal feeding’, ‘cow’s milk allergy’ and ‘functional constipation’. We also cross-referenced the articles that we retrieved for further studies.

## 3. Gastroesophageal Reflux

Gastroesophageal reflux (GER) refers to the retrograde movement of gastric contents into the esophagus, with or without regurgitation and vomiting [2]. This physiological process occurs routinely throughout the day in individuals of all ages. In healthy subjects, GER episodes are typically asymptomatic, fleeting, and last less than 3 min after meals [3]. It is estimated that approximately 50% of infants experience daily regurgitation, a transient and benign condition that typically resolves spontaneously by 12–15 months of age. Parental reassurance is crucial in managing uncomplicated cases, reducing the need for further investigation or intervention [2]. However, GER may culminate in gastroesophageal reflux disease (GERD), wherein the reflux leads to peptic-related esophageal complications and/or bothersome symptoms and signs affecting daily functioning, such as epigastric or chest pain, heartburn, faltering growth, hematemesis, respiratory manifestations, apnea, irritability, feeding difficulties, and iron deficiency anemia.

**Figure 1:**
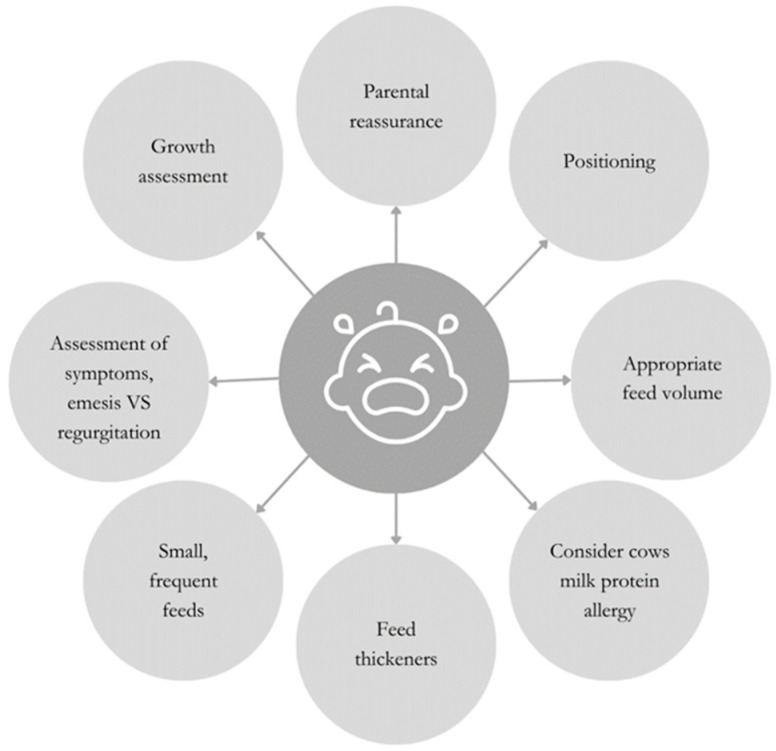
Behavioral and dietetic strategies to support the management of infantile GERD.

Conservative management, encompassing posture and dietary modifications, should be the primary approach to infantile GERD [4]. These approaches are summarized in Figure 1.

## 4. Postural Modifications

Postural modifications are widely used as an approach in the conservative management of infants with suspected GERD. Several studies have shown the efficacy of either left lateral position, prone position or head elevation in decreasing the total number of GER episodes in infants, irrespective of the medication given [5,6,7]. Nonetheless, caution is warranted due to compelling evidence associating prone and lateral sleeping positions with the increased risk of sudden infant death syndrome (SIDS). Both UK and international health services advocate for supine sleeping as the safest position to mitigate the risk of SIDS. Although elevating the head during supine positioning may appear intuitive, it poses the potential risk of infant rolling, thereby compromising the airway; hence, this practice is not recommended [8]. In contrast, for older children, alternative strategies such as head elevation or left lateral positioning may be considered [2] with the appropriate risk assessment.

## 5. Dietary Modification

### 5.1. Feeding Volume

There is consensus among professionals that overfeeding and excessive milk volumes heighten the risk of reflux episodes and GER. Consequently, it is recommended that infants be fed an appropriate volume of milk tailored to their gestational age and growth requirements. It is suggested that smaller, more frequent feeding may be advantageous in reducing gastric distension and thereby enhancing feed tolerance. However, it is worth noting that these recommendations stem from expert consensus rather than robust randomized controlled trials (RCTs).

### 5.2. Feed Thickeners

The utilization of pre-thickened feeds or adjunctive feed thickeners is a widely used approach for managing infants with GER or GERD and currently endorsed as a first-line treatment by the National Institute for Health and Care Excellence [9]. A variety of pre-thickened feeds or feed thickeners are available. While these products have been shown to decrease the occurrences of visible regurgitation, they do not reduce the actual number of reflux episodes, thus limiting their efficacy in managing GERD [10]. To date, there is no conclusive evidence to suggest the superiority of one thickening agent over another [8], although different products may be preferable in distinct clinical scenarios. For instance, in GERD-associated faltering growth, a maize-based supplement may be favored to provide additional calories.

### 5.3. Cow’s Milk Protein Allergy

Cow’s milk protein allergy (CMPA) and GERD are common complaints in infancy, and often present with overlapping symptoms. Several studies support the theory of a causal relationship between the two conditions, suggesting that GERD might be induced by CMPA at least in a subgroup of infants. Although it has been suggested CMPA underpins 40% of GERD, this association and its underlying mechanisms remain to be clarified [11]. Abnormal neuroimmune interactions induced by exposure to cow’s milk allergens might cause foregut dysmotility, which in turn result in severe GER and GERD [12]. Limited data exist regarding other common allergens such as egg, soy, and wheat.

Distinguishing between GERD and food allergy-related GERD is challenging due to the high overlap of symptoms. A thorough clinical history with a focus on allergies remains the primary approach for diagnosis. The presence of other atopic conditions, such as eczema, increases the likelihood of food allergy-related GERD. Additionally, if GERD fails to respond to first-line treatments, a therapeutic switch to an extensively hydrolyzed formula (EHF) or amino acid formula (AAF) or a maternal cows’ milk elimination diet can be attempted for 2–4 weeks, followed by reintroduction of cows’ milk protein to assess symptom improvement and potential recurrence retrospectively [2,13].

### 5.4. Feeding Difficulties

Feeding difficulties and aversive feeding behaviors are prevalent in GERD; feeding difficulties are particularly prominent when an allergic component is involved. The European Academy of Allergy and Clinical Immunology (EAACI) Task Force highlights that feeding difficulties occur in 13.6% to 40% of allergic cases [14] and manifest across a spectrum, from fussiness or picky eating to outright food refusal. A recent study found that preterm infants with untreated reflux exhibited significantly more feeding difficulties, including dysphagia and oral aversion, compared to those without reflux [15].

In severe cases where initial lifestyle interventions, medication management, or elimination diets have proven ineffective, the placement of an enteral feeding device may be necessary to ensure adequate nutritional intake. If tube feeding is required, encouraging oral intake, even in small amounts, can aid in transitioning back to oral autonomy at a later stage. Transpyloric feeding may be attempted if GERD persists as a significant issue; however, it has been shown that reflux can still occur, and events of aspiration and reflux-related hospital admissions remain possible [2].

### 5.5. Children and Adolescents

For older children and adolescents who are orally fed, dietary manipulation maybe advantageous for managing symptoms. Avoiding highly acidic food/beverages and limiting spicy foods may reduce direct esophageal irritation. Carbonated beverages may increase gastric distension, worsening symptoms; therefore, they should be minimized. Coffee, chocolate, and mint have been shown to reduce the LES tone and therefore should be limited if problematic. It is also important to consider eating behaviors by, for example, limiting late-night meals, as they can increase the gastric acid production. Large, calorie-dense meals increase gastric distension and may worsen symptoms [16]. Counselling from a dietitian can support an individualized dietary approach based on baseline diet.

## 6. Esophageal Atresia

Esophageal atresia (EA) is a common congenital anatomical malformation characterized by discontinuity of the esophagus with a prevalence of 2.4 to 3.2 per 10,000 live births worldwide [17]. Although survival rates have improved significantly with advancements in surgical techniques, long-term GI consequences remain problematic for many children, who present with ongoing GERD, dysphagia, feeding difficulties and esophageal strictures. The emerging literature also suggests an increased risk of allergic conditions, such as eosinophilic esophagitis (EoE) [18].

Following surgical repair, long-term eating and drinking difficulties are amongst the most significant issues seen in EA and can be due to multiple factors, such as late exposure to solids, esophageal dysmotility and feed-associated symptoms [19]. The latter include choking, dysphagia, slow eating and oral aversion. Feeding and nutrition are often emotive elements of daily living for parents, and difficulty at mealtimes is well documented to induce significant parental stress. Moreover, feeding anxiety, coupled with increased risk of choking and esophageal strictures in this patient group, might result in reduced parental QoL and an increased burden on health services [19,20]. This highlights the importance of early dietetic and speech and language therapy support to progress the diet safely, appropriately, and in a timely manner.

Underlying mechanisms for feeding difficulties include esophageal dysphagia, oropharyngeal dysphagia and aspiration, and aversion secondary to prolonged gastrostomy tube feeding [21]. Previous research has suggested that there is potentially a “critical window” for the introduction of certain flavors and textures, and missing this time period might increase the risk of aversive feeding later in childhood [22]. It has been shown that in children with EA, complementary feeding starts on average at 12 months of age compared to 4 months in the non-EA control group [23]. Furthermore, compared to healthy controls, children with EA have been shown more likely to refuse meals, eat slowly and cough/choke/vomit during meals [19]. These factors are associated with suboptimal nutritional intake and in turn with poor growth, which remains a long-term problem in patients with EA. An international cross-sectional study reported that children under 18 years had less than average height-for-age (HFA) and weight-for-age (WFA), with a median SD score of −0.41 and −0.63, respectively, compared to children without EA [19].

There are currently no published feeding guidelines for patients with EA, and advice is largely based on expert opinion and clinical experience. Furthermore, GERD is common in EA patients, and thus this advice would overlap with advice given for GERD. It is important to mention that EA patients are at higher risk of aspiration due to dysmotility and persistent GER. Krishnan et al. [23] reported between 14–40% of patients have respiratory symptoms, which include cough, wheezing and recurrent chest infections. Many of these patients undergo anti-reflux surgery when lifestyle modifications and medications are ineffective. Although the anti-reflux surgery is beneficial from a reflux management perspective, in some children, it can worsen feeding difficulties and dysphagia [24].

### 6.1. Sham Feeding

Before undergoing repair for long-gap EA, infants often undergo sham feeding, utilizing breast, bottle or solid feeding to aid in the development of oral feeding skills. Sham feeding facilitates the enhancement of swallowing abilities and the development of pharyngoesophageal coordination. This process involves the infant orally consuming milk or food, which is then extracted via a syringe connected to a tube in the upper pouch of the esophagus and subsequently recycled via gastrostomy [25]. It is crucial that the infant is awake, alert, and displaying hunger cues during sham feeding, as this supports the establishment of normal satiety regulation in young infants [26]. Tollne et al. [26] discovered that sham feeding has a positive impact on both parents and children.

### 6.2. Enteral Feeding

Early placement of a gastrostomy tube is a common practice in infants with EA while awaiting surgical intervention. Jejunal feeding may offer advantages in reducing the risk of aspiration and alleviating the burden of reflux and retching [21]. This approach could serve as an alternative to fundoplication, particularly in cases where there are concerns regarding esophageal dysmotility and the potential for esophageal stasis. Menzies et al. [27] observed that EA patients who underwent fundoplication experienced poorer outcomes in terms of growth compared to those who did not undergo the procedure.

### 6.3. Oral Feeding

Stewart et al. [28] described the impact of feeding a child with EA and highlighted the anxiety and stress experienced by the caregiver. Stresses include factors such as feeding outside the home, aspiration risk, and lack of transitioning through the feeding stages that would be expected in the normal weaning process. Multi-disciplinary support, including a dietitian and speech and language therapist, is fundamental to guide parents and help children in achieving their feeding milestones and navigating any barriers to feeding. Specific guidelines regarding oral feeding of the EA child are currently lacking, although charities have developed useful guidance to help parents with feeding their child. The IDDSI guidelines suggest progressing through textures as complementary feeding starts. IDDSI levels 3 and 4 are recommended as the appropriate first-line texture; these are considered liquidized and moderately thick substances with no lumps or solid pieces (they would not hold their form on a fork but would drip through the prongs (stage 3) or hold their form on a spoon (stage 4)). Examples include puréed vegetables, stage 1 baby jars, or smooth yoghurt. The texture between IDDSI type 3 and 4 can be manipulated by adding the baby’s usual milk as required. As with any child starting complementary foods, water should be introduced via a sippy cup or age-appropriate beaker. There is no need to thicken fluids, unless clinically advised. Encouraging sips of fluid whilst eating in children with EA can help with swallowing and food bolus transition. A recent systematic review identified 14 studies which highlighted the prevalence of using water for clearance and swallowing support with a rate of between 31–75% [29].

As the child progresses with their oral skills, the IDDSI texture can be increased, moving to stage 5, which refers to minced and moist substances. Any lumps present should be no thicker than 2 mm × 8 mm and should be easy to manipulate and squash with the tongue. Examples include quinoa, rice puff cereal or mashed vegetables. Finger foods are an important part of complementary feeding and termed transitional foods under the IDDSI criteria. These include bites and dissolve textures, i.e., those that start as a solid but change form when saliva is added. Once transitional foods are accepted and managed, IDDSI level 6 can be introduced; these should be soft and bite-sized and easily broken down with the back of a fork. The final texture progression is to IDDSI level 7; these are normal, everyday foods that are easy to chew. Gatzinsky and colleagues [30] report increased frequency of swallow difficulty as textures progress, with yoghurt being the least difficult to swallow and meat being the most.

Practical tips for preparation can be useful. These include the following:

- Grating hard-texture fruit and vegetables such as carrot or apple;
- Cutting meat against the grain or opting for moist cuts of meat, such as dark chicken meat or minced meats;
- Avoiding certain foods that are tricky to manage, especially those with a “doughy” or “claggy” texture, such as white bread, rusks, and banana;
- Removing tough skins on foods such as sausages;
- Adding sauces or gravy to food to provide additional moisture to aid swallowing;
- Using energy-rich fluids such as milk, fruit juice, soup, gravy and sauces to puréed food, rather than water, helps to maintain the nutritional value of the food offered.

In children with EA, the rate of texture progression may be delayed compared to those without. Typically, many children will not manage more textured foods until 18–24 months of age. Maybee and colleagues [31] reported results by age, identifying increased prevalence of swallowing difficulties in the first four years of life.

## 7. Achalasia

Achalasia is an esophageal disorder characterized by impaired peristalsis of the esophagus and an inability of the lower esophageal sphincter (LES) to relax [32]. The underlying etiology of achalasia is unknown, but it is believed to result from a loss of inhibitory neurons in the myenteric plexus of the distal esophagus and LES [33]. Patients often present with dysphagia for both solid and liquids, as well as chest pain, heartburn, reflux, regurgitation and respiratory symptoms. Weight loss and nutritional deficiencies are also common in both children and adults [34].

Nurko et al. [35] in a review including 475 children with achalasia found that 80% of patients presented with vomiting, 76% had dysphagia, 61% experienced weight loss, and 31% had faltering growth. Jarzebicka et al. [36] reported that a delay in the diagnosis of achalasia can lead to malnutrition, as only 20% of patients were able to tolerate a normal diet (with or without the need to drink fluids at mealtimes). There is no one diet that is recommended in achalasia as the tolerance of certain types of food seems to be individualized. The foods that may need to be avoided include those that are likely to cause an obstruction, that congeal in the esophagus or cause irritation, and/or foods that are either too hot or too cold. There are no formal evidence-based guidelines for eating in achalasia, and many practical suggestions have come from patients who live with the condition and who have contributed to forums. We would recommend a review by a registered dietitian who could collect a careful dietary history to identify any nutritional deficiencies. The dietitian may also be able help identify food types/textures that are causing concerns with regard to the patient’s swallow. The IDDSI framework may be helpful for guiding patients once suitable, well-tolerated textures are identified. Useful information regarding IDDSI can be found here: IDDSI Patient Handouts—https://iddsi.org/Resources/Patient-Handouts (accessed on 15 July 2024)—for achalasia and atresia.

For many patients with achalasia, a diet that is predominately liquid or a smooth purée in texture is required in the long term. Harder texture such as chips can increase the risk of esophageal perforation or food bolus obstruction (FBO).

If FBO occurs, previous case reports have suggested that carbonated drinks [37], especially Coca-Cola, have successfully dissolve the FBO [38,39] and could be considered in the long term as a preventative measure [39].

GERD is often present in achalasia following surgery; therefore, adopting dietary changes suggested above for the management of GERD may help to alleviate problematic symptoms.

## 8. Gastroparesis

Gastroparesis is motility disorder of the stomach characterized by delayed gastric emptying in the absence of mechanical obstruction [40]. It is associated with a wide non-specific spectrum of epigastric symptoms including nausea, early satiety, postprandial fullness, bloating and pain [41], which collectively cause challenges in nutritional intake and elevate the risk of malnutrition. Moreover, gastroparesis has significant negative impacts on the QOL of both patients and their family and imposes substantial healthcare costs.

While the etiology of gastroparesis in children is diverse, most cases are idiopathic, with the remaining resulting from drugs and GI surgery or less commonly poorly controlled diabetes and post-viral infections. The pathophysiology of gastroparesis is not fully understood but encompasses a variety of physiological alterations dependent on the underlying etiology. Proposed mechanisms include impaired fundal accommodation, altered gastric myoelectrical activity, diminished antral contractions, reduced interstitial cells of Cajal and compromised pyloric compliance.

Dehydration and malnutrition frequently complicate moderate-to-severe cases, with studies demonstrating compromised dietary intake and deficiencies in essential micronutrients such as vitamins D, E, K, folate, calcium, iron, magnesium, and potassium. Moreover, the routine use of proton pump inhibitors exacerbates micronutrient malabsorption, notably impacting iron and calcium absorption as reported in the literature [42].

Management strategies encompass a spectrum of interventions, including dietary modifications, optimization of glycemic control in diabetic patients, pharmacological therapies, and surgical therapies where indicated. In many cases, a multimodal therapeutic approach is required [43].

**Figure 2:**
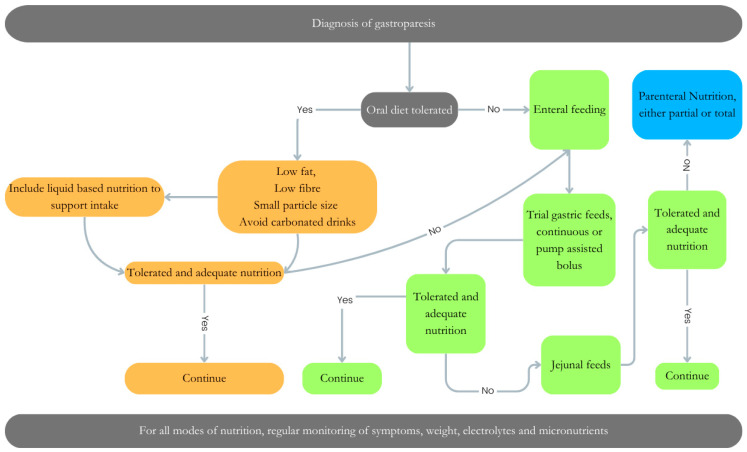
Dietary management of gastroparesis. Dietary management of gastroparesis will vary depending on the degree of disease severity. An Algorithm to support the decision/progression for oral diet (orange), enteral feeding (gastric Vs jejunal) (green) or parenteral nutrition (blue), or a combination of all nutritional approaches for management of a patient with gastroparesis.

Dietetic interventions play a pivotal role, via three primary modalities: oral feeding, enteral feeding (gastric or jejunal), and in severe cases, parenteral nutrition, tailored to alleviate symptomatic burden and optimize nutritional status (Figure 2).

Manipulating the oral diet should be the first approach in improving symptoms associated with gastroparesis. This may include “small particle size” diets, whereby foods that are well chewed, mashed with a fork, or broken down using a blender empty more readily from the stomach and reduce the need for gastric accommodation [44]. In addition, food timing and portion size can also play an important role. Patients with gastroparesis should be encouraged to adopt a “little and often” approach [45] and remain in an upright position post-prandially due to the association with facilitation of gastric emptying time [46].

Composition of the meal is also important to consider in gastroparesis. Meals high in fat and fiber are associated with delayed gastric emptying time; therefore, limiting these is often advised. Tolerance will vary between individuals and regular monitoring and support from a specialist dietitian is required to understand the appropriate amounts of such foods.

Where oral feeding is no longer tolerated, enteral feeding is a viable option. In the majority of cases in which enteral nutrition is required, jejunal feeding is recommended as it bypasses the affected location of the GI tract, improving feed tolerance [42]. In severe cases, PN maybe required; however, this should always be the last resort due to the high risk of complications, including mortality, associated with this mode of feeding.

## 9. Pediatric Intestinal Pseudo-Obstructive Disorders

Pediatric intestinal pseudo-obstructive (PIPO) disorders comprise the most severe motility conditions of the GI tract, characterized by the chronic inability of the gastrointestinal tract to propel its contents, mimicking mechanical obstruction. These are extremely rare conditions where a delay in diagnosis is common due to variations in clinical presentation and diagnostic methods. The clinical presentation depends on age of onset, location (“*the small bowel is always affected*”), and extent of severity and the disease phase (remission or relapse). During relapse, clinical manifestations such as significant abdominal distension, abdominal pain, vomiting and feed intolerance are seen; however, these can also be seen in the remission phase, making nutritional management challenging [47].

Nutrition-related issues are commonly seen in PIPO, with many children at presentation unable to maintain adequate growth and weight [47]. In addition, many patients report reduced QOL secondary to symptoms [48].

**Figure 3:**
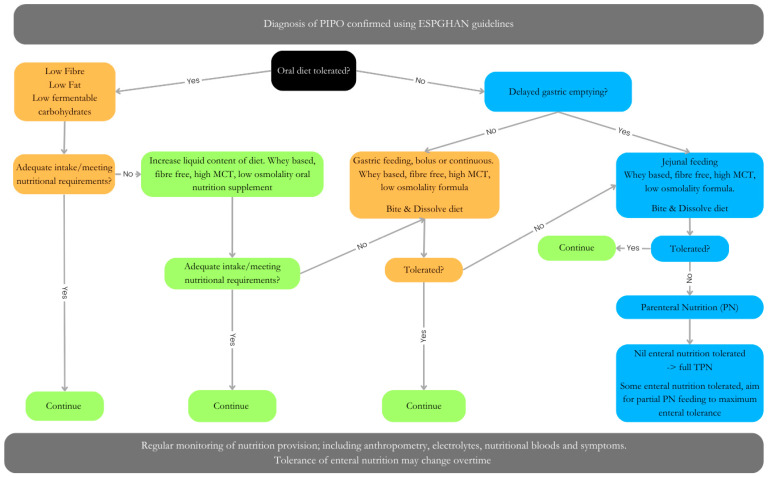
Dietary management of PIPO, adapted from Pescarin et al. [47]. Algorithm for dietary management of PIPO. Orange/green = no delated gastric emptying. Blue = delayed gastric emptying. Management may involve either oral, enteral (gastric Vs jejunal), parental nutrition (full Vs partial) or a combination of approaches. Patients tolerance may change over time.

The role of nutrition for gut function is well documented, with optimal nutrition improving GI motility and undernutrition or malnutrition worsening outcomes [49]. However, the ability to achieve optimal nutrition in PIPO is challenging and may require multiple approaches. This may include oral/sip feed or enteral feed administered as boluses or continuously by a nasogastric tube, gastrostomy and/or jejunostomy. If enteral feeding fails, parenteral nutrition (PN) is indicated. Between 60–70% of patients with PIPO require PN [50]. The nutritional approach should be tailored to the individual patient’s tolerance level (Figure 3). In addition to dietary manipulation, many children may also require ostomy (gastrostomy and/or ileostomy) formation to decompress the bowel as well as improve symptoms and feed tolerance. The latter is reported to occur in of 50% of cases [51].

Much of the literature to date is based on expert consensus as randomized control trials are limited in this patient group.

If safe and well tolerated, an oral diet should be encouraged where possible; however, careful consideration needs to be given to the macronutrient content, viscosity (liquid vs. solid), and frequency/schedule of eating. In children fed via the gastric route, it is known that several factors influence gastric emptying including protein type, amount and type of fat, energy density and osmolality (see above in gastroparesis). Manipulating these can help reduce symptoms seen in PIPO and improve QOL.

### 9.1. Fat

High-fat meals have been shown to delay gastric emptying, and therefore, advice to follow a low-fat diet is common practice in PIPO [47]. The specific type of fat should also be considered, with medium-chain triglyceride (MCT) fats shown to accelerate small bowel transit [52]. Therefore, an enteral formula with a higher MCT content maybe preferable in this patient group.

### 9.2. Fibers in PIPO

Fibers are categorized into soluble and insoluble, with each type impacting gut motility in differing ways. Soluble fibers (alginate, pectin, beta-glucane and polydextrose) have been shown to delay gastric emptying [53,54]. Insoluble fibers can lead of formation of a phytobezoar, which is a physical mass of indigestible plant material, posing the risk of mechanical obstruction. For this reason, it is advisable to avoid soluble fibers in PIPO patients with associated delayed gastric emptying, and insoluble fibers to prevent phytobezoar formation.

### 9.3. Carbohydrates

Although the exact impact of certain carbohydrates in PIPO is unknown, it is recommended to avoid fermentable carbohydrates FODMAPS (fermentable oligosaccharides including fructans [FOS] and galacto-oligosaccharides [GOSs], disaccharides [lactose], monosaccharides [fructose], and polyols) as their consumption could further exacerbate symptoms of abdominal distension and discomfort [47]. FODMAPs are rapidly fermented when they reach the proximal colon, leading to an increase in luminal organic acids, glycation of the end products, and lipopolysaccharides (LPSs).

A common issue in PIPO is small bowel bacterial overgrowth (SIBO) likely due to statis secondary to small bowel dysmotility. Although the mainstay treatment of SIBO is generally the administration of cyclical antibiotics [55], dietary manipulation such as a low-FODMAP diet might provide a sensible option to help reduce SIBO symptoms. Nevertheless, there are no data available specifically for this patient group.

### 9.4. Protein

It is recommended wherever possible that human milk is offered as a first line for infants, including those with neonatal onset PIPO. Breastmilk offers a variety of benefits that may be particularly useful in PIPO patients, including faster gastric emptying when compared to polymeric formula. If human milk is not available, then a whey-based extensively hydrolyzed formula may be recommended. There is some evidence that the formula milk that is 100% casein-based may increase the chances of bezoar formation [56]. In practice, it seems that hydrolyzed protein formulas seem to be better tolerated in our cohort of patients, even though the evidence to support this may be lacking. In older children and adults, it is recommended to initially trial a polymeric feed and only if not tolerated to move to either a hydrolyzed or amino acid-based feed. Unfortunately, there are no randomized controlled trials that analyze different formulas in children with PIPO, and thus the guidance is largely based on hypothesized views regarding gastric emptying, osmolality and the perceived likelihood of improved tolerance with these feeds.

### 9.5. Micronutrients

Due to the prolonged length to diagnosis and associated suboptimal nutrition, micronutrient deficiencies represent a risk in children with PIPO. Tang et al. [57] evaluated the micronutrient status of their PIPO cohort, demonstrating zinc and vitamin D deficiency were common. Interestingly, there was no significant difference in deficiencies at diagnosis compared to follow up. This demonstrates the need for regular nutritional monitoring by a specialist dietitian.

### 9.6. Meal Composition

The majority of patients with PIPO report exacerbation of symptoms with solid foods [58]. Digestible solids refer to those that can be broken down by the stomach and leave in liquid form. In children with PIPO and gastroparesis, liquid gastric emptying might be preserved, and hence a liquid diet is usually recommended if delayed gastric emptying is present. Thapar et al. [50] suggest that food that turns to liquid when mixed with saliva, termed “bite and dissolve”, may be an option in PIPO to enable taste stimulation and pleasure. However, the nutritional value of such foods is minimal; therefore, they are only suitable in combination with enteral or parenteral nutrition.

### 9.7. Enteral Tube Feeding

Where oral diet alone is not adequate for sustained nutrition, enteral tube feeding may be indicated to support or provide the sole source of nutrition. Manipulation of the feeding plan, be it bolus or continuous, may help improve feed tolerance. If severe gastroparesis for both solids and liquids is identified, jejunal feeding is recommended. The presence of the propagative phase III of the migrating motor complex (MMC) on antroduodenal manometry can help identify those patients who are more likely to tolerate jejunal feeding [59]. A small study in adults with CIPO has demonstrated the insertion of a gastro-jejunal feeding device reduced GI symptoms and thus improved nutritional status [49]. In these cases, the gastric port is used for sustained small bowel decompression and the jejunal port for delivery of enteral formula.

Oral diet, gastric and jejunal feeds should be tailored to the individual child. The support of a specialist dietitian is fundamental to manage nutrition in patients with PIPO.

### 9.8. Parenteral Nutrition

Approximately one-third of patients with PIPO require partial or total parenteral nutrition in the long term to either improve or maintain nutritional status [47]. Due to the high mortality and morbidity risk and high healthcare costs associated with PN, it should only be considered when all other nutrition routes have been exhausted. In terms of PN formulation and indication, ESPGHAN published detailed updated guidelines on pediatric parenteral nutrition in 2018 [60,61,62,63,64,65,66,67,68,69,70,71,72]. These guidelines remain relevant for PIPO patients as well as any other children presenting with intestinal failure.

Promisingly, the literature suggests that just under 50% of patients with PIPO who require PN are able to wean off over time [73]. Further research is required to understand if early decompression of the small bowel using either a gastrostomy for venting or ileostomy, improves long-term outcomes in PN dependency in PIPO.

## 10. Functional Constipation

Functional constipation (FC) is defined by constipation without an organic etiology, reported to impact between 5–27% of infants [74]. Moreover, for those who present with constipation, 95% of cases are attributed to FC [75].

As for other DGBIs, FC is diagnosed using The Rome IV age specific criteria. The etiology of FC is multifactorial, including a complex interaction between behavioral disorders (ADHD, austim), psychological disorders (anxiety, depression), lifestyle factors (diet, fluid intake, obesity, physical activity), stress and life events (abuse, trauma, stressors), parental factors (neuroticism, depression, overprotection), disruptive microbiota, genetics, colonic dysmotility and anorectal function [75].

The symptoms of FC include infrequent stool passage that is often associated with bloating, abdominal pain, and in some cases (particularly in children) fecal incontinence [75]. Infant dyschezia, another infant DGBI. is often confused with constipation due to symptoms associated with passing stool.

Table 1 demonstrates the difference between FC and infant dyschezia.

Although FC is the most common cause of constipation in pediatrics, red-flag symptoms should not be ignored and if present should be referred to a pediatric gastroenterologist for further investigation. Red-flag symptoms include any of the following [76]:

- Onset of symptoms from birth or within the first few weeks of life;
- Delayed passage of meconium (>48 h after birth);
- Ribbon stools;
- Neurological involvement such as leg weakness;
- Abdominal distension with bilious vomiting;
- Bladder involvement.

Although medical management using laxative therapy remains the cornerstone of treatment for FC, lifestyle modifications including postural changes, dietary (fiber, fluids) and physical activity level can have an significant impact on symptoms. These may be considered first-line approaches or in combination with medical management.

### 10.1. Fibers in Constipation

Fibers are a complex carbohydrate found in plant-based foods; undigested, they pass into the bowel, where they have 3 main mechanisms through which they impact intestinal motility. Fiber in the diet accelerates colonic transit by increasing stool bulk and produces short-chain fatty acids (butyrate, propionate, acetate), which increase osmotic load and either indirectly or directly change the intraluminal microbiome by altering luminal pH [77].

Although a diet lacking in fiber is associated with an increased risk of constipation [78], there is limited evidence in the pediatric population that additional fiber supplementation or intake above normative values is beneficial to improve outcomes. Several trials have assessed the efficacy of fibers as a therapeutic modality for constipation in children. These include psyllium, cocoa husk, glucomannan, partially hydrolyzed guar gum, FOS, GOS and a mixture of fibers. However, the results of different systematic reviews and metanalysis report either no efficacy or the marginal success of the use of fibers in the treatment of constipation in children [79,80,81]. Guidelines from ESPGHAN/NSPGHAN recommend a normative fiber intake through a whole-food-based diet where possible [82]. Fiber requirements vary depending on a child’s age. Under 1 year of age, there are no fiber recommendations; however, from 12 months onward, fiber intake should be expressed as “age plus 5–10 g” [77].

Fiber-rich foods include fruits, vegetables, whole grains, oats, pulses, nuts and seeds [83]. From a practical perspective, it may not always be achievable for a child to meet their fiber requirements; this may be due to mode of feeding (enteral formula) or aversive/selective feeding behaviors. In these circumstances, addition of a fiber supplement might be beneficial to meet normative requirements.

As discussed, fruits and vegetables are high in dietary fiber. It has been shown in adults with FC that certain fruits, particularly the addition of two kiwi fruits (without skin), significantly increase complete spontaneous bowel movements by 1.5 times per week. Improved associated GI symptoms, such as abdominal pain, nausea and bloating were also reported [84]. It has been hypothesized that the unique cell structure of the kiwi allows for its high capacity for absorbing water, which increases water retention in the small bowel and ascending colon and in turn increases total colonic volume. Kiwi also contains substantial amounts of the protease actinidin, which has the potential to activate protease-activated receptors (PARs). The latter are found on enterocytes, lymphocytes, and enteric nerves and play a significant role in visceral sensitivity [85]. Although studies to date are based on adults, the option to include kiwi in a child’s diet offers an additional dietary option for managing constipation as well as providing essential micronutrients such as vitamin C and other antioxidants.

### 10.2. Fluid

There is an association between low fluid intake and increased risk of constipation [86]. There have been no benefits found in research regarding increasing fluid intake above normal requirements [82]. The National Institute of Health and Care Excellence [87] guidelines have provided recommendations for water intake for children (Table 2).

### 10.3. Probiotics

Patients with FC have been found to have an altered microbiome when compared to healthy controls [88]. It is reported in the literature that children with FC may have dominant levels of Lactobacillus spp and lower levels of Bacteroides when compared to healthy children [87]. The pathophysiology for this dysbiosis is not well understood, and probiotics have been proposed as a treatment option for FC, aiming to modulate the microbiome [86]. The potential benefits of optimizing the microbiome in FC include metabolite and fermentation products, which lead to an osmotic impact and gas production, as well as modulating colonic pH, which all are hypothesized to improve gastrointestinal motility [86]. ESPGHAN/NASPGHAN have concluded that the evidence does not currently support the use of probiotics. There is limited evidence from one study that may show a benefit of the use of symbiotics [89]. Further research is needed to examine whether probiotics could be used as a future treatment for FC.

### 10.4. Posture

**Figure 4:**
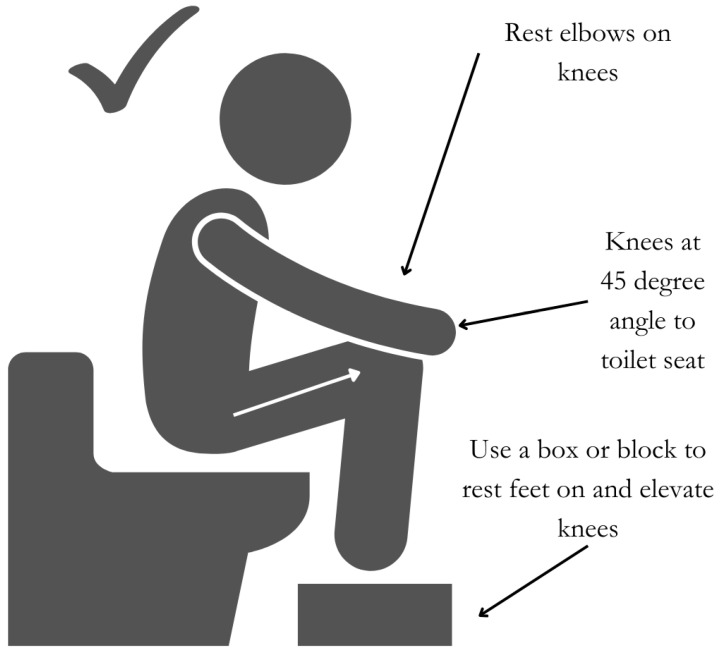
Optimal position for stool passage.

The position of the body when passing stool is an important factor to ensure adequate and optimal defecation. Ensuring a position in which the angle between the longitudinal axis of the anal canal and the posterior rectal line is parallel to the longitudinal axis of rectum, termed the ano-rectal angle, helps facilitate stool expulsion [86]. In this position, the knees should be elevated above the hips at 45-degree angle to the toilet seat using a block or step, and the elbows should be placed on the knees in a forward-leaning position (Figure 4).

Timing of toileting can improve ease. A child should be encouraged to sit on the toilet for up to 5 min, one to three times a day, following meals and first thing in the morning to take advantage of the gastrocolic reflex [86].

### 10.5. Physical Activity

Decreased physical activity has been identified as a possible risk factor for constipation [90]; however, there are limited RCTs that have looked specifically at the impact of functional constipation on physical activity [82]. Adult studies have demonstrated an increase in transit time following physical activity, although the frequency of bowel evacuation was not impacted [91].

Regular physical activity has multiple documented health benefits regardless of the impact on FC in children, and therefore healthcare professionals should advise regular physical activity in line with government guidelines.

### 10.6. Allergy

A causal relationship between food allergy (FA) and functional constipation has been highly debated in recent decades. By reviewing the available literature, in a recent paper, the European Academy of Allergy and Clinical Immunology (EAACI) has acknowledged non-immunoglobulin E (IgE)-mediated FA as potential cause of chronic constipation at least in a subgroup of children unresponsive to optimal dietary, lifestyle, and stool softeners [92]. The rate of efficacy of either cow’s milk free diet or more restricted diets in children with functional constipation unresponsive to stool softeners has been reported to range between 28 and 78% [93]. Cow’s milk is the most common allergen involved in food allergy-related constipation, although other allergens such as wheat, soya, and egg have also been reported.

Although the underlying mechanisms by which food allergy induces constipation remain undefined, abnormal neuroimmune interactions have been suggested to play a key pathophysiological role. Intense lymphocytic and eosinophilic infiltration in the rectal mucosa, responding to elimination diet, has been described in children with chronic constipation related to cow’s milk allergy [94,95]. Borrelli et al. [96] demonstrated that children with FA-related chronic constipation had both an increased rectal mast cell density and an increased number of mast cells in close proximity to submucosal rectal nerve endings, which correlated with anorectal motor abnormalities measured via anorectal manometry. Both histological and anorectal motor abnormalities reversed following an 8-week period of exclusion of cow’s milk, egg, and soy proteins.

As with other non-IgE-mediated food allergies, the process of dietary elimination for 2–8 weeks followed by reintroduction should be used to confirm/exclude an atopic element to constipation. In breast-fed infants, this will require a maternal elimination, and for those infants who are formula-fed, a change from whole protein to a hypoallergenic formula is recommended. Support from a specialist dietitian to guide this process is highly recommended.

## 11. Conclusions

Motility disorders often present challenges for diagnosis and management. Management strategies for these conditions encompass a range of interventions, including medical therapies, surgical procedures, psychological interventions, dietary adjustments, or a multimodal approach when necessary. Early intervention is crucial to mitigate long-term complications such as growth failure and feeding difficulties. For all forms of motility disorders, nutritional management requires a tailored approach to improve symptoms, QOL and nutritional status. This may include dietary modifications, enteral feeding strategies, and, in severe cases, parenteral nutrition (or a combination of all of the above).

Advancements in understanding the underlying mechanisms of GI motility disorders and refining their diagnostic and treatment modalities offer hope for improved outcomes and QOL for affected individuals. Collaboration among healthcare professionals, including dietitians, speech and language therapists, and gastroenterologists, remains paramount in delivering comprehensive care and optimizing nutritional outcomes for pediatric patients with these conditions.

## Figures and Tables

**Table 1:** Comparison of symptoms seen in FC and infant dyschezia; information tabulated based on Zeevenhooven et al. [74].

Symptoms	Functional Constipation	Infant Dyschezia
Behavior before passing stool	Distress on stooling/straining Bleeding associated with hard stool	Straining/crying/turning red in the face with effort
Type of stool passed	Hard large stool Rabbit droppings BSC * Type 1–3	Soft stool
Frequency of stooling	Fewer than three complete stools per week	Usually daily (eventually)
Onset of symptoms	First few weeks of life (not from birth)	First few months of life
Treatment	Dietary interventions Osmotic laxative treatment	Resolves spontaneously within a few weeks

* BSC = Bristol stool chart.

**Table 2:** Pediatric fluid requirements per day.

Age	Total Fluid per Day (mL)
0–6 months	700 (breastmilk/formula milk)
7–12 months	800 (milk and complementary foods and beverages)
1–3 years	1300
4–8 years	1700
Boys: 9–13 years	2400
Girls: 9–13 years	2100
Boys: 14–18 years	3300
Girls: 14–18 years	2300

Total water intake per day, including water contained in food (mL).
